# Cytotoxic activity of methanolic fractions of different *Marrubium* spp. against melanoma cells is independent of antioxidant activity and total phenolic content

**DOI:** 10.1002/2211-5463.12755

**Published:** 2019-12-02

**Authors:** Malgorzata Kozyra, Agnieszka Korga, Marta Ostrowska, Ewelina Humeniuk, Grzegorz Adamczuk, Renata Gieroba, Anna Makuch‐Kocka, Jaroslaw Dudka

**Affiliations:** ^1^ Department of Pharmacognosy with Medicinal Plant Laboratory Medical University of Lublin Poland; ^2^ Independent Medical Biology Unit Medical University of Lublin Poland; ^3^ Department of Toxicology Medical University of Lublin Poland

**Keywords:** antioxidant activity, free phenolic acid, *in vitro* cytotoxicity, *Marrubium*, melanoma

## Abstract

The *Marrubium* genus (horehound) has proved to be an abundant source of biologically active compounds, but there is little knowledge about its potential anticancer activity. Moreover, some *Marrubium* species have not been the subject of study in this regard. In this study, we performed comparative analysis of phenolic acid (PhA) content and total phenolic content in fractions obtained from methanolic extracts of *Marrubium vulgare* L. (common horehound), *Marrubium cylleneum* Boiss. & Heldr. and *Marrubium friwaldskyanum* Boiss herbs. We examined the cytotoxicity of these fractions against a human melanoma cancer cell line (A375) and normal human skin fibroblasts (BJ) using a 3‐(4,5‐dimethylthiazol‐2‐yl)‐2,5‐diphenyl‐tetrazolium bromide test, cell cycle analysis and real‐time monitoring of cell viability. We detected caffeic, *p*‐coumaric, ferulic and gentisic acids among the PhAs. Although the extracts obtained demonstrated low total phenolic content and did not show significant antioxidative properties, the nonhydrolyzed PhA fraction exhibited cytotoxic activity against a human melanoma cancer cell line, without affecting normal fibroblasts. Both acidic and alkaline hydrolysis abolished this activity, indicating that the esterified forms of phenolic compounds caused the observed cytotoxic effects. Further investigation of these compounds may facilitate the development of novel drugs for cancer treatment.

## Graphical abstract

Nonhydrolyzed phenolic acid fractions obtained from three species of *Marrubium* genus revealed similar composition and weak antioxidative activities. However, we demonstrate that they have selective cytotoxic activities against a human melanoma cancer cell line, but lack activity against normal fibroblasts. Both acidic and alkaline hydrolysis abolished this activity, which indicates that the esterified forms of phenolic compounds caused the observed effects.

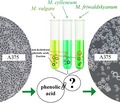

**Abbreviations**
AA: antioxidant activity
AAI: AA index
DPPH: 2,2‐diphenyl‐1‐picrylhydrazyl
FA: free PhA
FB: PhAs after acidic hydrolysis
FC: PhAs after alkaline hydrolysis
GAE: gallic acid equivalent
MTT: 3‐(4,5‐dimethylthiazol‐2‐yl)‐2,5‐diphenyl‐tetrazolium bromide
SD: standard deviation
TPC: total phenolic content

*Marrubium *(*Lamiaceae*) is a genus of around 50 species of flourishing plants native to Europe, Asia, North Africa and South America that has been known in folk medicine since ancient times. *Marrubium vulgare* is the best known and characterized species. It has been used to treat a number of diseases, including digestive problems, cough and any infectious diseases. At present, it seems to be underestimated; however, recently, this plant has been considered as a source of biologically active compounds for the formulation of new medicinal products and substrates in the cosmetic industry. A number of biological activities of *M. vulgare* have been described. It showed hypoglycemic 1, analgesic 2, 3, antiphlogistic 4, antihypertensive 5, 6 and antioxidant activities, 7, 8 and much more 9. In the group of active compounds among *Marrubium* species, diterpenoids, sterols and phenylpropanoids (including caffeic acid derivatives and flavonoids) have been reported 10, 11, 12. Despite the wide range of phytochemical components 10, most of the earlier described activities attribute to diterpenoid lactone marrubiin, which is present in high concentrations 13. Fewer reports concern cytotoxic effects of *Marrubium* genus extracts against cancer cells 14. Diterpene compounds exhibit anticancer activity 15; however, according to the the PubChem BioAssay database for Marrubiin 16, marrubiin did not show any cytotoxic effect against 66 tested cancer cell lines.

Plants are an invaluable source of compounds with potential anticancer effects. Many of them are increasingly being used in the treatment of cancer or are being evaluated in clinical trials (e.g. vincristine, vinblastine, doxorubicin, dacarbazine, bleomycin and cyclophosphamide) 17. There is a belief that the antineoplastic properties of different plant compounds are associated with antioxidant activity (AA) that correlates with phenolic content 18. Phenolic acids belong to an important group of phenolic compounds. This group includes substances that contain a phenolic ring and at least one organic carboxylic functional group. Phenolic acids may be present in plants as esters or glycosides conjugated with other natural compounds such as flavonoids, alcohols, sterols and glucosides 19.

The mechanisms of antineoplastic action of phenolic acids (PhAs) are not yet well described. It is suspected that they involve free radicals scavenging and activation of metabolic enzymes. They are also supposed to regulate gene expression and modulate cellular signalling pathways 20.

Biological activities and PhA content of many species other than *M. vulgare* have not been well described yet. Therefore, the aim of our research was the comparison analysis of the composition of PhAs in fractions obtained from 70% methanol extract of *M. vulgare* L. and lesser known species *Marrubium cylleneum* Boiss. & Heldr. and *Marrubium friwaldskyanum* Boiss, as well as the assessment of their antioxidant and cytotoxic properties.

## Materials and methods

### The plant material

Flowering herbs of *Marrubium* genus (127.0 g) were collected in July on a sunny day about 9:00 a.m. in the Medicinal Plant Garden of the Department of Pharmacognosy and Medicinal Plant Unit (Voucher specimens: 05‐07/2016; Lublin, Poland). The herbs were dried at 45 °C, powdered and sieved (sieves 0.315 and 0.074 mm). The procedure of preparation follows the conditions of the Polish Pharmacopoeia VI.

### The extraction procedure

**Figure 1:**
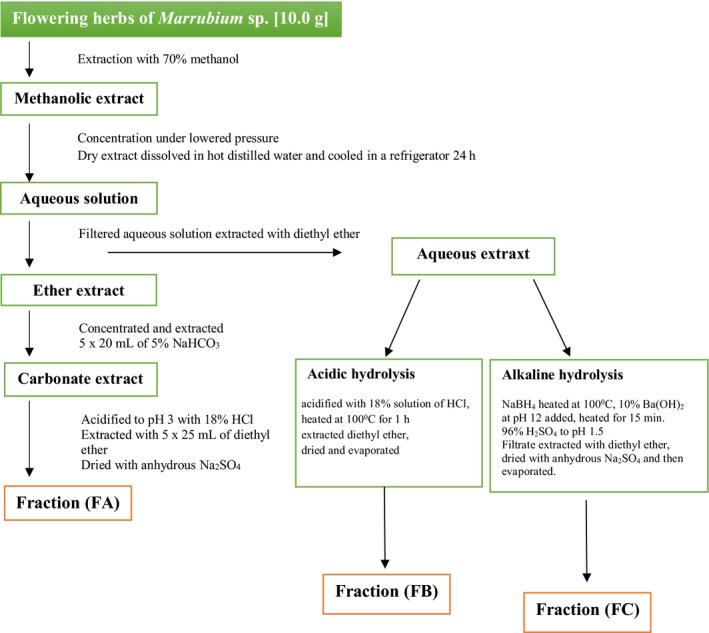
The extraction procedure of three polyphenolic fractions: FA, FB and FC of the tested *Marrubium* genus.

The extraction procedure was shown on Fig. 1. The plant material (10 g) was placed in the stainless steel cell of Dionex (Sunnyvale, CA, USA) ASE 100 with 70% methanol 99.8% pure p.a. basic (Polish Reagents, Lublin, Poland) at boiling point 78 °C as solvent. The extraction conditions were as follows: temperature, 85 °C; flush volume, 80%; purge time, 100 s; number of cycles, 3; and cycle duration, 10 min. Then extracts were evaporated under lowered pressure and analyzed following the instructions of Ibrahim and Towers’s method 21. The dry extract was dissolved in 30 mL of hot distilled water, then cooled in a refrigerator for 24 h. Next, ballast substances were removed by quantitative disc filters (Filtrak, Thermalbad Wiesenbad, Germany) filtration. Next, filtrates were extracted with 5 × 30 mL of diethyl ether pure p.a. (Polish Reagents). Obtained ether and aqueous extracts were subjected to PhA analysis with the method described by Ibrahim and Towers and Schmidtlein and Hermann 21, 22. As a result of these procedures, three polyphenolic fractions, free PhAs (FA), PhAs after acidic hydrolysis (FB) and PhAs after alkaline hydrolysis (FC), were obtained for each tested *Marrubium* genus. For HPLC analysis, fractions FA, FB and FC were dissolved in 90% methanol, filtered with 0.45‐μm polytetrafluoroethylene membrane filters (Cronus Syringe Filter, PTFE, SMI‐LabHut Ltd, Churcham, Gloucestershire, UK) and transferred to 10‐mL calibrated vials.

### PhA content

The content of PhAs in flowering *Marrubium* sp. herbs was specified by Arnov’s method (Specol Helios Beta, UniCam, Birmingham, UK, λ = 490 nm, Arnov’s reagent) and then calculated. Three grams of the plant material was extracted with 50 mL of 100% methanol on water bath (at a boiling extractant temperature) for 30 min. Each extract was filtered, and the plant material was re‐extracted twice with a fresh portion of solvent. The obtained methanolic extracts were evaporated to dryness and dissolved in 20 mL of hot water. After cooling, extracts were filtered and filled up with water. Five milliliters of water, 1 mL of extract, 1 mL of hydrochloric acid (18 g·L^−1^), 1 mL of Arnov’s reagent and 1 mL of sodium hydroxide solution (40 g·L^−1^) were added to the studied extracts and filled up with water. The absorbance was measured after 20 min. The contents of PhAs were calculated as caffeic acid according to the formula: *X* = *A *× 3.5087/*m*, where *A* is the measured absorbance of the sample, and *m* is the weight of the plant substance and was expressed as % of caffeic acid (Sigma‐Aldrich, St. Louis, MO, USA) 22, 23. All tests were performed in triplicate.

### The RP‐HPLC analysis

Compounds were separated on a 250 × 4.6‐mm stainless steel column Hypersil XDB‐ C18 (Agilent, Santa Clara, CA, USA), packed with 5 μm particles using a stepwise mobile phase gradient prepared from 1% aqueous acetic acid (component A) and methanol (component B; v/v). The gradient was as follows: 0 min, 5% component B into A; 5 min 20% component B into A; 20 min 35% component B into A; 35 min 55% component B into A; and 45 min 75% component B into A. The mobile phase flow rate was 1 mL·min^−1^, the sample injection volume was 10 μL and the elution was performed at 25 °C. Liquid chromatography pumps, autosampler, column oven and diode array detector were monitored and controlled by hp chem station rev.10.0 software (Agilent). Retention times were compared with standards using UV spectra (λ = 254 nm for benzoic acid derivatives: protocatechuic, *p*‐hydroxybenzoic, vanillic and syringic acids; 280 nm for gallic acid; 325 nm for cinnamic acid derivatives: *p*‐coumaric ferulic, caffeic and chlorogenic acids) as a comparative parameter. Following PhAs, standards were used: *p‐*hydroxybenzoic, *m*‐hydroxybenzoic, protocatechuic, gallic, vanillic, syringic, *trans‐*cinnamic, *p‐*coumaric, caffeic, ferulic, rosmarinic, chlorogenic, coumaric, *o*‐coumaric, isovanillic and gentisic acids (Sigma‐Aldrich).

### Determination of the total phenolic content in the plant extracts

Total phenolic content (TPC) of the extract from *Marrubium* sp. was determined using Folin–Ciocalteu reagent according to the method described previously 24. One hundred microliters of each extract (*c* = 3.52 mg·mL^−1^) was combined with 100 μL Folin–Ciocalteu reagent (Sigma‐Aldrich) and 800 μL of 20% sodium carbonate (Na_2_CO_3_) aqueous solution (0.075 g·mL^−1^). The mixture was shaken immediately at room temperature, and 20 min later the absorbance was measured at 760 nm. To prepare the curve, we measured the absorbance of different concentrations of gallic acid (0.005, 0.01, 0.02, 0.04, 0.06, 0.08 and 0.10 µg·mL^−1^) following the procedure described earlier. The TPC concentration was calculated as gallic acid equivalent (GAE) per 1 mg of each extract using the following equation obtained for the GAE calibration curve: sample *A* = 0.0067 × gallic acid (µg) + 0.0132, *R* ^2^ = 0.9864. All tests were performed in triplicate.

### Evaluation of the AA

AA was determined spectrophotometrically in line with the method of Brand‐Williams *et al*. 25 using the methanolic solution of 2,2‐diphenyl‐1‐picrylhydrazyl (DPPH) radical (85.00 µg·mL^−1^). Dilutions of *Marrubium* extracts were made to obtain the concentrations of 0.125, 0.188, 0.250, 0.375, 0.500, 0.625, 0.750, 0.875, 1.000, 1.125 and 1.258 mg of referred plant substance per 1 mL. The diluted extracts were mixed with 3.9 mL of DPPH methanolic solution. The mixture was shaken and left to stand for 30 min in the dark at room temperature. Next, the absorbance was measured spectrophotometrically at 517 nm. The inhibitory concentration (IC_50_) is defined as the quantity of antioxidant that inhibits DPPH radical formation by 50%. To compare the scavenging properties of investigated extracts, we used the IC_50_ value for gallic acid (0.352 mg·mL^−1^), caffeic acid (0.341 mg·mL^−1^), rutin (1.215 mg·mL^−1^) and Trolox (0.825 mg·mL^−1^). Triplicate measurements were made and the radical scavenging activity was counted using the formula: % Inhibition = [(*A*b − *A*a)/*A*b] × 100, where *A*b is the absorption of a blank sample, and *A*a is the absorption of the testing extract solution. The results are presented as mean ± standard deviation (SD). The lack of normalization of DPPH results restricts the comparison of the antioxidant strength of pure compounds and plant extracts. According to Scherer and Godoy 26, we calculated the AA index (AAI) considering the mass of both DPPH and tested compounds in the chemical reaction as follows: AAI = concentration of DPPH (µg·mL^−1^)/IC_50_ (µg·mL^−1^). In line with Scherer and Godoy, AAI values less than 0.5 attest to low AA, values of 0.5–1.0 attest to moderate activity, values of 1.0–2.0 attest to a strong activity and values greater than 2.0 attest to very strong AA 26.

### The cell culture

The research was conducted on the human melanoma cell line (A375; ATCC, Manassas, VA, USA) and skin fibroblast cell line (BJ; ATCC). The cells were cultured with RPMI‐1640 and Eagle’s minimal essential medium, respectively (PAA Laboratories, Pasching, Austria), supplemented with 10% FBS (Life Technologies, Carlsbad, CA, USA) in standard conditions: 37 °C and 5% CO_2_.

### The cytotoxicity analysis

The cytotoxicity of tested extracts was measured using the 3‐(4,5‐dimethylthiazol‐2‐yl)‐2,5‐diphenyl‐tetrazolium bromide (MTT) Cell Proliferation Assay Kit (Life Technologies). FA, FB and FC fractions obtained from *M. vulgare*,* M. friwaldskyanum* and *M. cylleneum* were added to the cell cultures in the following final concentrations: 200, 100, 50, 25 and 12.5 µg·mL^−1^. Further measurement was performed as previously described 27. One percent Triton X‐100 was used as a positive control for the MTT assay. IC_50_ values were calculated by regression analysis in MS Excel (Microsoft Corp., Redmond, WA, USA).

### The real‐time monitoring of cell viability

The xCELLigence DP analyzer (Acea Biosciences, San Diego, CA, USA) was used to monitor the real‐time cell viability. The analyzer uses electrical impedance as the readout. Its level depends on the size, shape and number of cells and their adhesion to the plate. A375 cells were treated with FA fractions of three tested *Marrubium* sp. in their log phase (12 h) in final concentrations of 200, 100 and 50 µg·mL^−1^. The assessment was carried out for the following 24 h in quadruplicates.

### The cell cycle analysis

After 24 h of incubation, the tested cells were collected and analyzed according to the manufacturer’s protocol. The test involves staining with 4′,6‐diamidino‐2‐phenylindole, which binds to double‐stranded DNA of cells. To analyze the DNA content of each cell cycle stage, we used the NucleoCounter NC‐3000 (ChemoMetec, Allerod, Denmark). One percent Triton X‐100 was used as a positive control.

### Statistical analysis

The obtained data were given as mean ± SD and analyzed by statistica 12 software (StatSoft, Krakow, Poland). The statistical significance was evaluated by Student’s *t*‐test; *P ≤ 0.05, **P ≤ 0.01 and ***P ≤ 0.001 were considered statistically significant.

## Results

### The TPC in plant extracts

**Figure 2:**
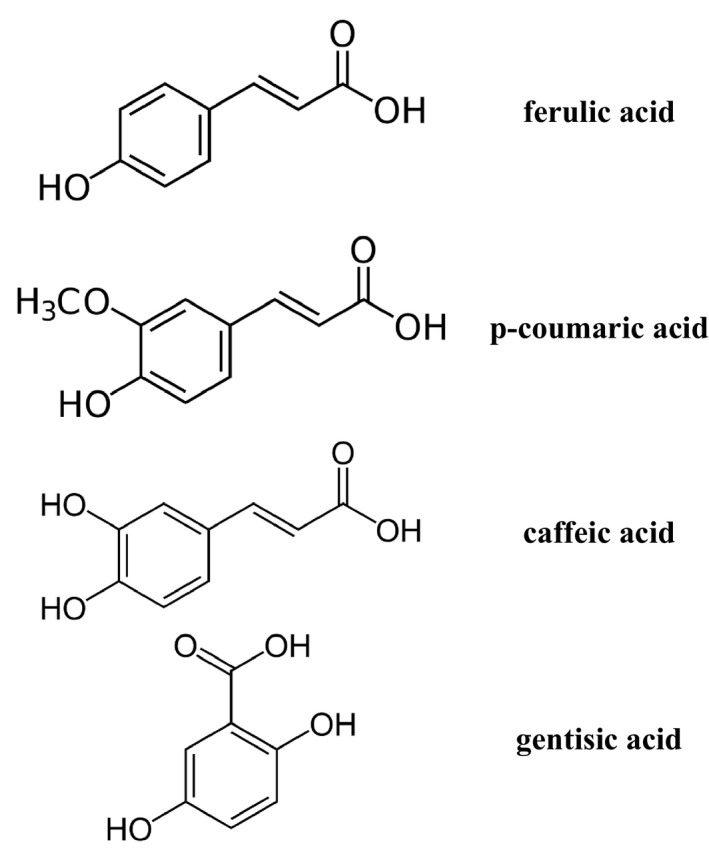
The structures of identified gentisic, caffeic, *p*‐coumaric and ferulic acids using HPLC analysis.

The PhA content differed among tested species. Especially rich in PhAs were aerial parts of *M. vulgare* (0.65%), whereas *M. friwaldskyanum *(0.32%) and* M. cylleneum* (0.22%) were characterized by lower content of phenolic compounds (Table 1). In this article, we also analyzed the content of PhAs in fractions FA, FB and FC using the RP‐HPLC method. We identified gentisic, caffeic, *p*‐coumaric and ferulic acids (Fig. 2). Gentisic acid was present only in fraction FB. The results are presented in Table 2. The composition of identified PhA content was similar. We identified ferulic, *p*‐coumaric and caffeic acids in nonhydrolyzed PhAs fraction (FA). After acidic hydrolysis (FB), gentisic acid occurred, which was absent after alkaline hydrolysis (FC). The mean amount of TPC in methanolic extracts of *M. vulgare*, *M. friwaldskyanum* and *M. cylleneum* was as follows: TPC 19.00, 18.22 and 18.09 mg GAE·g^−1^, respectively (see Table 1).

**Table 1:** The total content of PhAs in tested extracts was determined spectrophotometrically (λ = 490 nm) and given as mean absorbance (*A*) ± SD. The content of PhA was expressed as % of caffeic acid. The TPC values were given as GAEs (mg of GAE·g^−1^ of extract).

Marrubium species	A	PhA (%)	TPC (mg GAE·g^−1^)
M. vulgare	0.560 ± 0.031	0.65	19.00
M. friwaldskyanum	0.187 ± 0.023	0.32	18.22
M. cylleneum	0.271 ± 0.011	0.22	18.09

**Table 2:** Identified PhAs in fractions FA, FB and FC of methanol extracts of tested *Marrubium* sp. using the RP‐HPLC method.

PhA	*M. vulgare*			*M. cylleneum*			*M. friwaldskyanum*		
	FA	FB	FC	FA	FB	FC	FA	FB	FC
Gentisic	−	+	−	−	+	−	−	+	−
Caffeic	+	+	+	+	+	+	+	+	+
*p*‐Coumaric	+	+	+	+	+	+	+	+	+
Ferulic	+	+	+	+	+	+	+	+	+

### DPPH radical scavenging activity

IC_50_ values were presented as a concentration that halved the amount of DPPH radicals (µg·mL^−1^). The largest capability for neutralizing DPPH radicals was noticed for *M. vulgare* and *M. friwaldskyanum* at 570 and 590 μg·mL^−1^ (Table 3), respectively. Lower activity was noted for *M. cylleneum* extract, 760 μg·mL^−1^. Table 3 shows the obtained IC_50_ values and results of the AAI for extracts and pure compounds. In terms of assumed classification, studied gallic acid had the highest value of AAI, followed by caffeic acid, Trolox and rutin, because they are proven antioxidants. The *M. vulgare*, *M. cylleneum* and *M. friwaldskyanum* extracts obtained with methanol solvent had a very low AAI (0.149, 0.112 and 0.144, respectively), suggesting weak AA.

**Table 3:** IC_50_ of the DPPH radicals (µg·mL^−1^; *c*_DPPH_ = 85.00 µg·mL^−1^) and AAI of tested extracts and reference pure substances.

Extract/pure solution	IC_50_	AAI
Methanol extract from *M. vulgare*	570.00	0.149
Methanol extract from *M. cylleneum*	760.00	0.112
Methanol extract from *M. friwaldskyanum*	590.00	0.144
Caffeic acid	4.55	18.681
Gallic acid	3.95	21.519
Rutin	14.5	5.862
Trolox	10.5	8.095

### The cytotoxicity analyses

**Figure 3:**
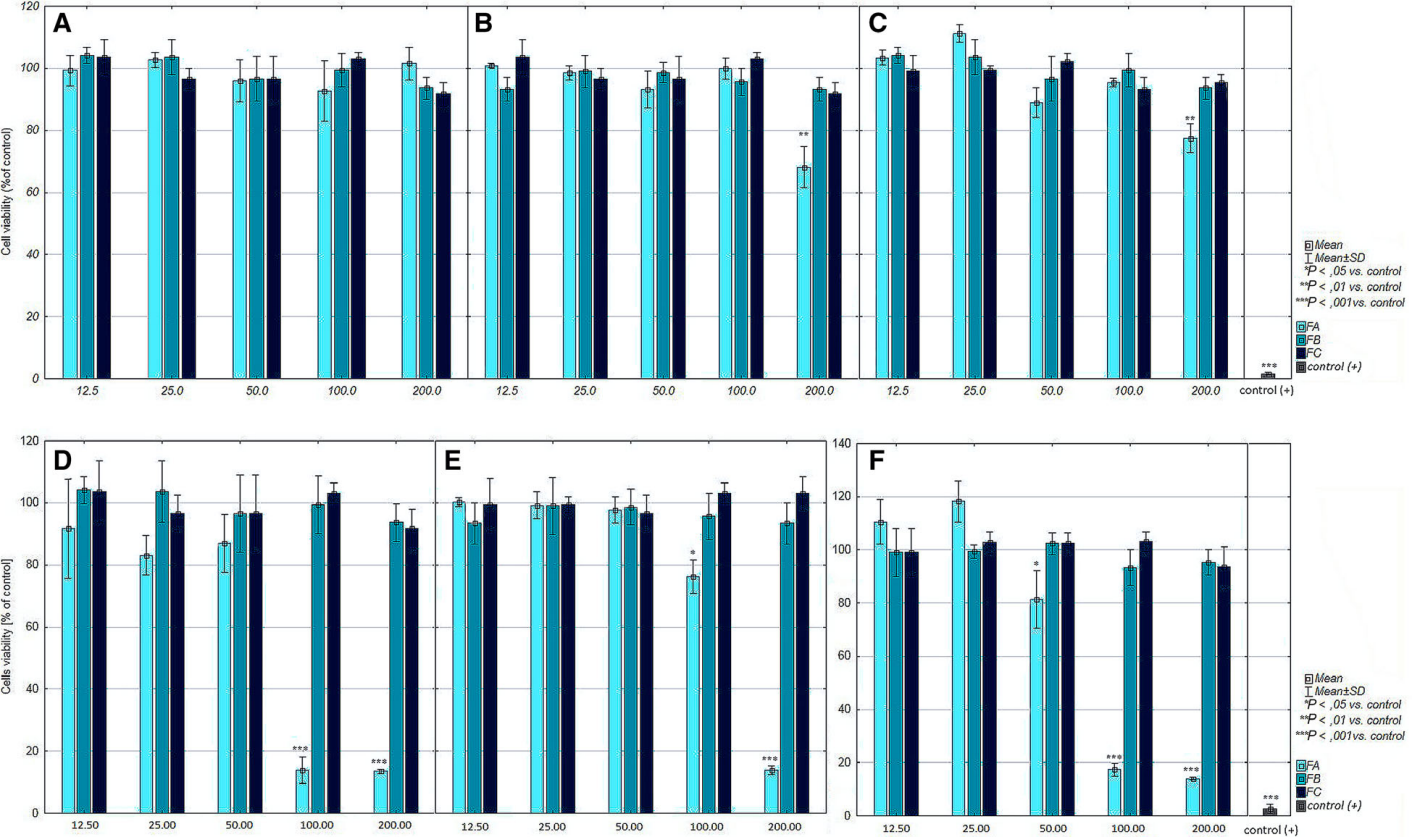
The cytotoxic activity of *M. vulgare* (A), *M. cylleneum* (B) and *M. friwaldskyanum* (C) FA, FB and FC fractions on human skin fibroblast cell line and *M. vulgare* (D), *M. cylleneum* (E) and *M. friwaldskyanum* (F) FA, FB and FC fractions on human melanoma cancer cell viability, measured after 24 h using MTT assay. One percent Triton X‐100 was used as a positive control. Data are presented as the mean ± SD of experiment triplicates. The statistical significance of differences between control and treated culture was evaluated by Student’s *t*‐test and presented as *P ≤ 0.05, **P ≤ 0.01 and ***P ≤ 0.001.

**Figure 4:**
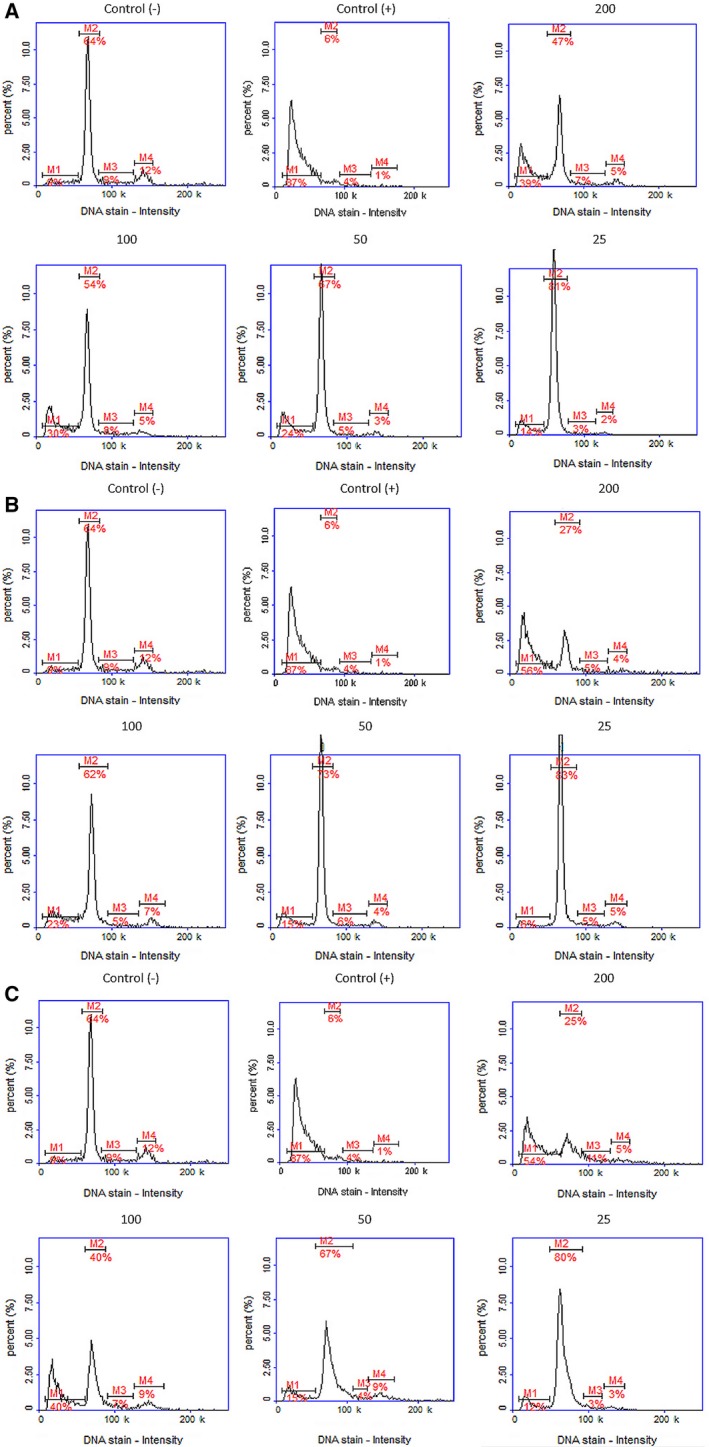
Cell cycle analysis of human melanoma cancer cells during treatment with (A) *M. vulgare*, (B) *M. cylleneum* or (C) *Marrubium friwaldskyanum* FA fractions at concentrations of 200, 100, 50 or 25 µg·mL^−1^.

**Figure 5:**
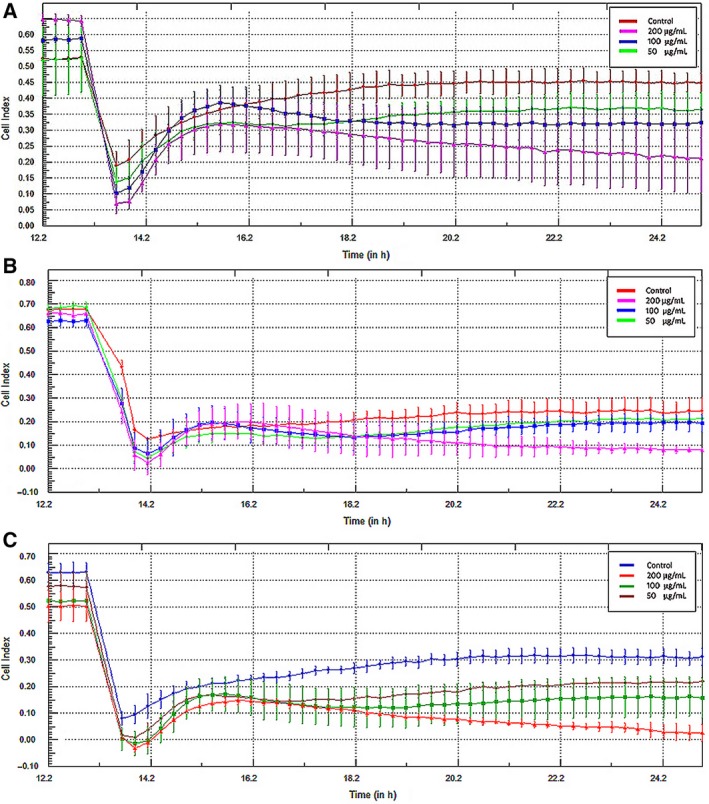
The real‐time monitoring graph of cell viability after treatment with 50, 100 and 200 µg·mL^−1^ of fraction A from methanol extract of *M. vulgare* (A), *M. cylleneum* (B) and *M. friwaldskyanum* (C).

The MTT test results revealed slight toxicity of FA fraction of *M. cylleneum *and* M. friwaldskyanum* on normal cells (viability lowered to 68.2 ± 11.5% and 77.5 ± 8.16%). The remaining extracts showed no significant cytotoxic activity (Fig. 3A–C). In the case of cancer cells, the MTT test results revealed that only the FA fraction of all tested species had cytotoxic activity against the A375 cell line. In all tested species, the FA fraction significantly decreased cell viability (Fig. 3D–F). In the case of *M. vulgare* and *M. friwaldskyanum*, this activity was similar (IC_50_ = 74.16 and 75.49 µg·mL^−1^, respectively), and the *M. cylleneum* FA fraction had weaker cytotoxic activity (IC_50_ = 142.03 µg·mL^−1^). The cell cycle analysis confirmed these observations. Changes in histograms looked similar for the extracts of all three species; with increase in the extract concentration in culture (50–200 µg·mL^−1^), an increase in the peak corresponding to the sub‐G1 phase (apoptotic cells) was observed, along with a decrease in the G1 phase peak. Furthermore, it has been shown that all extracts in concentration of 25 µg·mL^−1^ caused cell cycle arrest in the G1 phase: the population of cells in G1 phase increased from 64% to 81%, 83% and 80% for *M. vulgare*, *M. cylleneum *and* M. friwaldskyanum*, respectively, without important changes in the G2/M phase (Fig. 4A–C). The real‐time monitoring partially confirmed the earlier observation. Obtained growth curves showed that only extracts with a concentration of 200 µg·mL^−1^ had a strong cytotoxic effect. However, all tested *Marrubium* sp. inhibited cell growth at 100 and 50 µg·mL^−1^ concentrations (Fig. 5A–C).

## Discussion

Today’s medicine benefits from plants of the *Marrubium* genus. The content of some species and their biological activities are already well known, such as *M. vulgare*, which is used in digestive and respiratory systems diseases. There are few reports concerning the cytotoxic effect of *Marrubium* genus extracts on cancer cells 11, 14, 28, and this activity is attributed to different groups of compounds. Antineoplastic properties of different plants may be associated with the content of phenolic compounds including PhAs 18. No report addresses whether PhAs or their conjugates originated from *Marrubium* genus plants as compounds contributing to the cytotoxic activity.

Generally, crude extracts include a lot of constituents, which may express antioxidant and cytotoxic activities. For this reason, simple fractionation of *Marrubium* sp. extracts was carried out to separate phenolic compounds and find out the most potent fraction. Methanol brings the phenolic compounds out from plant matter with the best efficiency 29. The focus of our research was PhAs incorporated in methanolic extracts of *Marrubium* plants, as well as their antioxidant and cytotoxic activity against cancer and normal cells.

The fractions of FAs of *M. vulgare*, *M. cylleneum* and *M. friwaldskyanum* were compared. The quality content of the composition of the studied extracts turned out to be allied. We identified ferulic, *p*‐coumaric, caffeic and gentisic acids. *M. vulgare* showed the highest content in terms of quantity of PhAs. Analysis of hydrolyzed fractions demonstrated that there must have been a conjugated form of gentisic acid in nonhydrolyzed fraction A that absolved during acidic hydrolysis, and free gentisic acid occurred in fraction B. To our knowledge, gentisic acid conjugates, as active ingredients, had not been published yet.

The *Marrubium* genus is regarded as essentially rich in phenolic compounds, among other phytochemicals. However, obtained extracts demonstrated low TPC and did not show significant antioxidative properties that are believed to be crucial for a plant’s therapeutic features. The antioxidant properties of PhAs are associated with phenolic hydroxyl groups connected with ring structures 30. Interestingly, antioxidative properties of *M. vulgare* were associated with hepatoprotective 31 and anticancer activity 32.

Current data on the relationship between the polyphenol content of plants with their AA are contradictory. Such a high correlation between the two has been observed 33, whereas other authors have found no such correlation or only a very weak one 30. The results of our research showed that the AA of methanolic extracts was low, and the TPC was considered low. It is surprising in light of foregoing reports dealing with the *Marrubium* genus. The proper clarification of observed discrepancies may be found in the research of Bouterfas *et al*., in which *M. vulgare* plants derived from three geographical regions of Algeria were studied 34. During extraction, researchers used solvents at various polarities. TPC and flavonoids levels ranged between 40.7 and 160 mg GAE·g^−1^ and 27.4 and 66.3 mg catechin equivalents·g^−1^, respectively, depending on the origin of the plant. Therefore, substantial differences were observed. Moreover, the lowest value in the study by Bouterfas *et al*. 34 was more than twice as high as the TPC value obtained in our research for all studied *Marrubium* spp. Based on the IC_50_ of Bouterfas *et al*. 34, which ranged from 33.7 to 774 μg·mL^−1^, our extracts may be classified as ones with the poorest antioxidative properties. Regardless of weak antioxidative potential, tested nonhydrolyzed extracts demonstrated similar cytotoxic activity on the human melanoma cancer cell line and was weak on normal cells. Moreover, in our research, we have shown that lower concentrations of the tested extracts caused the cell cycle arrest in the G1 phase in a dose‐dependent manner. These results indicate that the constituents of the extracts may cause DNA damage or in some other way they are blocking the transition to phase.

Hydrolyzed extracts did not show any antiproliferative activities. Therefore, cytotoxic properties may be put down to PhAs conjugated with other natural compounds. The majority of PhAs are linked through ester, ether or acetal bonds either to structural components of the plant (e.g. cellulose, proteins or lignin) or to larger polyphenols, or smaller organic molecules (e.g. glucose) 35, 36. The importance of these compounds for the activity of plant extracts is underestimated.

## Conclusions

Studied fractions of FAs demonstrated similar content in three *Marrubium* sp. Regardless of low TPC value and weak antioxidative activities, cytotoxic properties of nonhydrolyzed PhA fraction on the human melanoma cancer cell line were demonstrated, whereas no activity against normal cells was observed. Both acidic and alkaline hydrolyses abolished this activity. Because of the large amount of possible combinations of acid connections, one should take extensive studies up to determine the active compounds contained in the tested extracts. In general, studies dealing with antiproliferative properties of *Marrubium* genus plants report whole groups of compounds, diterpenes, flavonoids and phenylpropanoids, as the active components of extracts. There is a lack of experiments showing properties of a single compound or synergy of particular compounds, as well as their mechanism of action, what comes across as essential in the implementation of *Marrubium* genus plant extracts into cancer treatment. In our studies, we showed that the cytotoxic effect may be caused by esterified forms of PhAs, which should be investigated in further studies.

## Conflict of interest

The authors declare no conflict of interest.

## Author contributions

MK conceived and supervised the study, and carried out the research. AK contributed to the formation of the experimental concept and wrote the first draft of the manuscript. MO contributed to the formation of the experimental concept, carried out the research and revised the manuscript. EH assisted in the research work and contributed to analysis of the data. GA assisted in the research work and contributed to analysis of the data. RG designed the protocol and carried out the research. AM‐K assisted in the research work and contributed to analysis of the data. JD revised the manuscript.

## References

[1] Roman RR , Aharcon AF , Lara LA and Flores SJL (1992) Hypoglycemic effect of plants used in Mexico as antidiabetics. Arch Med Res 23, 59–64. 1308793

[2] De Souza MM , DeJesus R , Cechinel‐Filho V and Schlemper V (1998) Analgesic profile of hydroalcoholic extract obtained from *Marrubium vulgare* . Phytomed 5, 103–107. 10.1016/S0944-7113(98)80005-6 23195761

[3] Meyre‐Silva C , Yunes RA , Schlemper V , Campos‐Buzzi F and Cechinel‐Filho V (2005) Analgesic potential of marrubiin derivatives, a bioactive diterpene present in *Marrubium vulgare* (Lamiaceae). Farmaco 60, 321–326. 15848207 10.1016/j.farmac.2005.01.003

[4] Fathiazad F , Rameshrad M , Asghari S , Hamedeyazdan S , Garjani A and Maleki‐Dizaji N (2017) Phytochemical screening and anti‐inflammatory effect of *Marrubium vulgare* L. Methanol extract on carrageenan‐induced paw inflammation in rats. Pharm Sci 23, 3–11.

[5] El Bardai S , Lyoussi B , Wibo M and Morel N (2001) Pharmacological evidence of hypotensive activity of *Marrubium vulgare* and *Foeniculum vulgare* in spontaneously hypertensive rat. Clin Exp Hypertens 23, 329–343. 11349824 10.1081/ceh-100102671

[6] El Bardai S , Lyoussi B , Wibo M and Morel N (2004) Comparative study of the antihypertensive activity of *Marrubium vulgare* and of the dihydropyridine calcium antagonist amlodipine in spontaneously hypertensive rat. Clin Exp Hypertens 26, 465–474. 15554450 10.1081/ceh-200031818

[7] Orhan IE , Belhattab R , Şenol FS , Gülpinar AR , Hoşbaş S and Kartal M (2010) Profiling of cholinesterase inhibitory and antioxidant activities of *Artemisia absinthium*, *A. herba‐alba, A. fragrans, Marrubium vulgare, M. astranicum, Origanum vulgare* subsp. glandulossum and essential oil analysis of two *Artemisia* species. Ind Crop Prod 32, 566–571.

[8] Pukalskas A , Venskutonis PR , Salido S , De Waard P and Van Beek T (2012) Isolation, identification and activity of natural antioxidants from horehound (*Marrubium vulgare* L.) cultivated in Lithuania. Food Chem 130, 695–701.

[9] Villanueva RJ and Esteban MJ (2016) An insight into a blockbuster phytomedicine; *Marrubium vulgare* L. Herb. More of a myth than a reality? Phytother Res 30, 1551–1558. 27271209 10.1002/ptr.5661

[10] Meyre‐Silva C and Cechinel‐Filho V (2010) A review of the chemical and pharmacological aspects of the genus *Marrubium* . Curr Pharm Des 16, 3503–3518. 20942795 10.2174/138161210793563392

[11] Argyropoulou A , Samara P , Tsitsilonis O and Skaltsa H (2012) Polar constituents of *Marrubium thessalum* Boiss. & Heldr. (Lamiaceae) and their cytotoxic/cytostatic activity. Phytother Res 26, 1800–1806. 22407803 10.1002/ptr.4654

[12] Kozyra M , Biernasiuk A , Antonik R , Malm A and Zgórka G (2018) The phytochemical and antimicrobial examination of phenolic acids contained in extracts from four *Marrubium* species. Acta Pol Pharm 75, 937–950.

[13] Popoola O , Elbagory A , Ameer F and Hussein A (2013) Marrubiin. Molecules 18, 9049–9060. 23899837 10.3390/molecules18089049 PMC6269822

[14] Lodhi S , Vadnere G , Sharma V and Usman M (2017) *Marrubium vulgare* L.: A review on phytochemical and pharmacological aspects. J Complement Med Res 6, 429–452.

[15] Islam MT (2017) Diterpenes and their derivatives as potential anticancer agents. Phytother Res 31, 691–712. 28370843 10.1002/ptr.5800

[16] National Center for Biotechnology Information . PubChem Compound Database; CID=73401. https://pubchem.ncbi.nlm.nih.gov/compound/73401 (accessed Oct. 24, 2018).

[17] Habtemariam S and Lentini G (2018) Plant‐derived anticancer agents: lessons from the pharmacology of geniposide and its aglycone, genipin. Biomedicines 6, 39. 10.3390/biomedicines6020039 PMC6027249 29587429

[18] Hamedeyazdan S , Sharifi S , Nazemiyeh H and Fathiazad F (2014) Evaluating antiproliferative and antioxidant activity of *Marrubium crassidens* . Adv Pharm Bull 4(Suppl1), 459–464. 25364663 10.5681/apb.2014.068 PMC4213786

[19] Herrmann K (1989) Occurrence and content of hydroxycinnamic and hydroxybenzoic acid compounds in foods. Crit Rev Food Sci Nutr 28, 315–347. 2690858 10.1080/10408398909527504

[20] Rosa LS , Silva NJA , Soares NCP , Monteiro MC and Teodoro AJ (2016) Anticancer properties of phenolic acids in colon cancer a review. J Nutr Food Sci 6, 468.

[21] Ibrahim R and Towers G (1960) The identification, by chromatography, of plant phenolic acids. Arch Biochem and Biophys 87, 125. 13852593 10.1016/0003-9861(60)90132-6

[22] Schmidtlein H and Hermann K (1975) Quantitative analysis for phenolic acids by thin layer chromatography. Chromatography 115, 123.

[23] Szaufer‐Hajdrych M (2004) Phenolic acids in leaves of species of the *Aquilegia* genus. Herb Pol 50, 10–14.

[24] Singleton V and Rosi J (1965) Colorimetry of total phenolics with phosphomolybdic–phosphotungstic acid reagents. Am J Enol Viticult 16, 144–158.

[25] Brand‐Williams W , Cuvelier M and Berset C (1995) Use of free radical method to evaluate antioxidant activity. LWT 28, 25–30.

[26] Scherer R and Godoy HT (2009) Antioxidant activity index (AAI) by the 2,2‐diphenyl‐1‐picrylhydrazyl method. Food Chem 112, 654–658.

[27] Korga A , Józefczyk A , Zgórka G , Homa M , Ostrowska M , Burdan F and Dudka J (2017) Evaluation of the phytochemical composition and protective activities of methanolic extracts of *Centaurea borysthenica* and *Centaurea daghestanica* (Lipsky) Wagenitz on cardiomyocytes treated with doxorubicin. Food Nutr Res 61, 1344077. 28747863 10.1080/16546628.2017.1344077 PMC5510226

[28] Karioti A , Skopeliti M , Tsitsilonis O , Heilmann J and Skaltsa H (2007) Cytotoxicity and immunomodulating characteristics of labdane diterpenes from *Marrubium cylleneum* and *Marrubium velutinum* . Phytochemistry 68, 1587–1594. 17475293 10.1016/j.phytochem.2007.03.027

[29] Stanković MS (2011) Total phenolic content, flavonoid concentration and antioxidant activity of *Marrubium peregrinum* L. extracts. J Sci 33, 63–72.

[30] Terpinc P , Polak T , Šegatin N , Hanzlowsky A , Ulrih NP and Abramovič H (2011) Antioxidant properties of 4‐vinyl derivatives of hydroxycinnamic acids. Food Chem 128, 62–68. 25214330 10.1016/j.foodchem.2011.02.077

[31] Akther N , Shawl AS , Sultana S , Chandan BK and Akhter M (2013) Hepatoprotective activity of *Marrubium vulgare* against paracetamol induced toxicity. J Pharm Res 7, 565–570.

[32] Yamaguchi K , Liggett JL , Kim NC and Baek SJ (2006) Anti‐proliferative effect of horehound leaf and wild cherry bark extracts on human colorectal cancer cells. Oncol Rep 15, 275–281. 16328068 PMC2440569

[33] Piluzza G and Bullitta S (2011) Correlations between phenolic content and antioxidant properties in twenty‐four plant species of traditional ethnoveterinary use in the Mediterranean area. Pharm Biol 49, 240–247. 21323476 10.3109/13880209.2010.501083

[34] Bouterfas K , Mehdadi Z , Elaoufi MM , Latreche A and Benchiha W (2016) Antioxidant activity and total phenolic and flavonoids content variations of leaves extracts of white Horehound (*Marrubium vulgare* Linné) from three geographical origins. Ann Pharm Fr 74, 453–462. 27553439 10.1016/j.pharma.2016.07.002

[35] Klick S and Herrmann K (1998) Glucosides and glucose esters of hydroxybenzoic acids in plants. Phytochemistry 27, 2177.

[36] Lam TBT , Kadoya K and Liyama K (2001) Bonding of hydroxycinnamic acids to lignin: ferulic and p‐coumaric acids are predominantly linked at the benzyl position of lignin, not the aˆ‐position, in grass cell walls. Phytochemistry 57, 98. 10.1016/s0031-9422(01)00052-8 11423145

